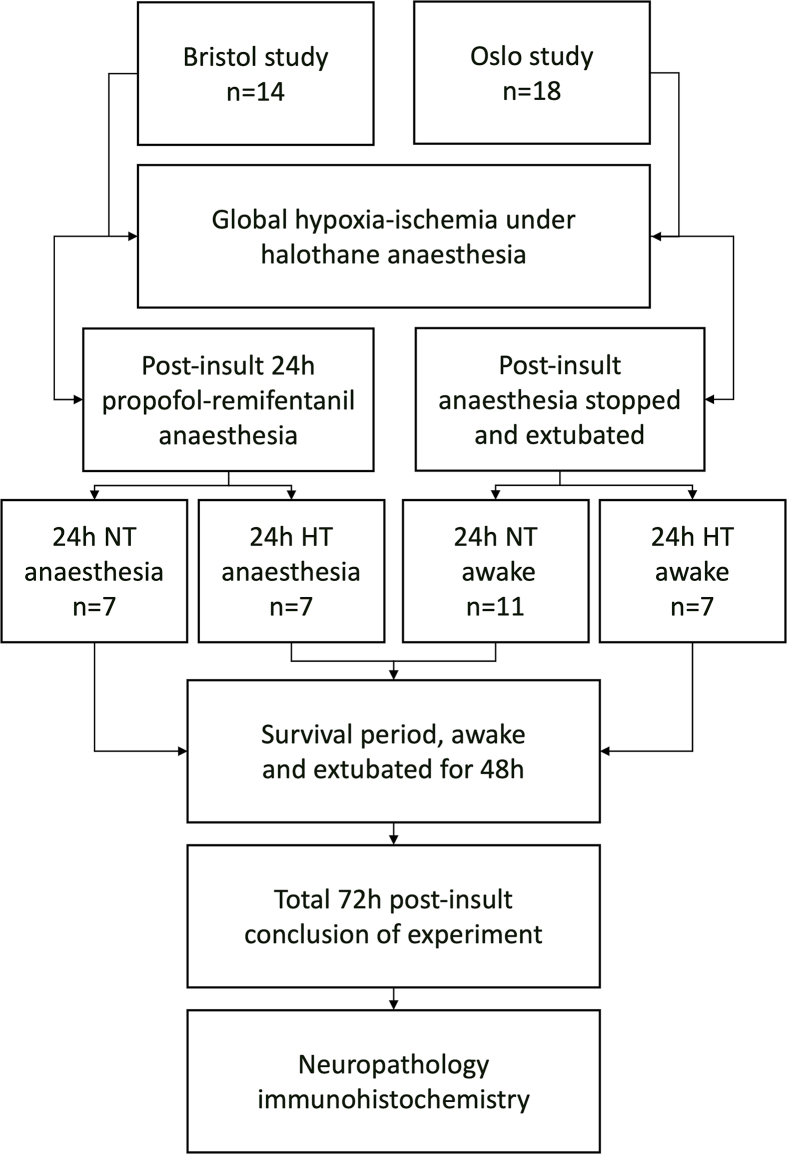# The effects of anaesthesia on cell death in a porcine model of neonatal hypoxic-ischaemic brain injury

**DOI:** 10.1016/j.bjao.2024.100283

**Published:** 2024-05-05

**Authors:** Julia K. Gundersen, Ela Chakkarapani, David A. Menassa, Lars Walløe, Marianne Thoresen

**Affiliations:** 1 Department of Physiology, Institute of Basic Medical Sciences, University of Oslo, Oslo, Norway; 2 Department of Neurology, Akershus University Hospital, Lørenskog, Norway; 3 Translational Health Sciences, St. Michael's Hospital, Bristol Medical School, University of Bristol, Bristol, UK; 4 Department of Neuropathology and The Queen's College, University of Oxford, Oxford, UK; 5 Department of Women's & Children's Health, Karolinska Institutet, Solna, Sweden

**Keywords:** apoptosis, caspase 3, cerebellum, hypoxia-ischemia, hippocampus, hypothermia, neonatal, Purkinje cells

## Abstract

**Background:**

Hypothermia is neuroprotective after neonatal hypoxic-ischaemic brain injury. However, systemic cooling to hypothermic temperatures is a stressor and may reduce neuroprotection in awake pigs. We compared two experiments of global hypoxic-ischaemic injury in newborn pigs, in which one group received propofol–remifentanil and the other remained awake during post-insult hypothermia treatment.

**Methods:**

In both studies, newborn pigs were anaesthetised using halothane during a 45-min global hypoxic-ischaemic insult induced by reducing *F*io_2_ and graded hypotension until a low-voltage <7 μV electroencephalogram was achieved. On reoxygenation, the pigs were randomly allocated to receive 24 h of normothermia or hypothermia. In the first study (*n*=18) anaesthesia was discontinued and the pigs' tracheas were extubated. In the second study (*n*=14) anaesthesia was continued using propofol and remifentanil. Brain injury was assessed after 72 h by classical global histopathology, Purkinje cell count, and apoptotic cell counts in the hippocampus and cerebellum.

**Results:**

Global injury was nearly 10-fold greater in the awake group compared with the anaesthetised group (*P*=0.021). Hypothermia was neuroprotective in the anaesthetised pigs but not the awake pigs. In the hippocampus, the density of cleaved caspase-3-positive cells was increased in awake compared with anaesthetised pigs in normothermia. In the cerebellum, Purkinje cell density was reduced in the awake pigs irrespective of treatment, and the number of cleaved caspase-3-positive Purkinje cells was greatly increased in hypothermic awake pigs. We detected no difference in cleaved caspase-3 in the granular cell layer or microglial reactivity across the groups.

**Conclusions:**

Our study provides novel insights into the significance of anaesthesia/sedation during hypothermia for achieving optimal neuroprotection.

Therapeutic hypothermia is neuroprotective after neonatal hypoxic-ischaemic encephalopathy, as demonstrated in various animal models^1^ and clinical trials.^2^ The effects of hypothermia have been thoroughly explored in large animal models of hypoxic-ischaemic encephalopathy regarding optimal temperature^3^ and duration^4^ of cooling, the effect of delayed onset,^5^, ^6^, ^7^ concurrent inflammation^8^ and stress.^9^ Among large animal models, the newborn pig is an excellent species for studying hypoxic-ischaemic injury and hypothermia as it has similar brain morphology to humans, including gyrencephaly, comparable brain growth spurt,^10^ similar white matter distribution,^1^ and comparable cardiovascular responses.^11^

Despite the successful translation of hypothermia into clinical practice, there are significant differences in the treatment benefit from hypothermia. The number needed to treat to reduce death or major neurodevelopmental disability is estimated to be 7.^2^ Concurrent drug use in the neonatal intensive care unit, including sedation and analgesia, is believed to be among the many factors thought to influence neuroprotection.^12^ Cooling to below normothermia is a stressor for most species, resulting in increased plasma cortisol concentration in unanaesthetised newborn pigs^9^ and prolonged cortisol release in unanaesthetised fetal lambs.^13^ Thoresen and colleagues^9^ reported neuroprotection to be abolished after 24 h of immediate hypothermia in unsedated pigs, whereas consistent neuroprotection was demonstrated in anaesthetised newborn pigs after hypothermia for 3 h,^14^ 12 h,^15^ and 24 h.^16^ Moreover, we recently reported neuroprotection to be abolished in immature rats subjected to restraint stress and discomfort from a rectal thermometer probe.^17^ In preterm infants, the number of painful procedures is associated with decreased cerebellar volume^18^ and cortical thickness^19^ at school age. This evidence raises concern for infants with hypoxic-ischaemic encephalopathy undergoing hypothermia, likely prompting clinicians to administer increasing doses of opioids during cooling.^12^

The anaesthetic and sedative agents administered during hypothermia in the newborn pig may independently influence injury susceptibility and neuroprotection.^20^ Propofol and remifentanil, the anaesthesia cocktail commonly used in these models, are suggested to exert antiapoptotic and neuroprotective effects.^21^, ^22^, ^23^ Fentanyl, a different opioid, was reported to induce apoptosis in the cerebellar granular cell layer,^24^ but not the cerebrum^25^ in newborn pigs. Overall, the available evidence suggests anaesthetic and sedative agents to have differing effects on cell death in the newborn brain.

With this study, we aimed to conduct a comparative analysis of two historical experiments of global hypoxic-ischaemic injury in newborn pigs. Our surviving pig model integrates clinically relevant brain damage, a therapeutic standard of clinical care including post-insult seizures, multiorgan injury, and cell resolution neocortical neuropathology.^26^ We compare two studies in which all pigs received halothane-induced anaesthesia during the hypoxic-ischaemic insult. In one study, the anaesthesia was switched to propofol–remifentanil during the 24 h of normothermia or whole-body hypothermia^15^; whilst in the other study, the pigs remained awake throughout the normothermia or the cooling period.^9^ Based on previously published findings,^9^ we hypothesised that the administration of anaesthesia would enhance hypothermia neuroprotection in this model.

## Methods

### Experimental design

The study in which pigs were anaesthetised throughout the hypoxic-ischaemic insult and treatment was conducted in Bristol (UK) and approved by the University of Bristol Ethical Review Panel.^15^ The study in which pigs were awake during subsequent treatment was conducted in Oslo (Norway) and approved by the Norwegian Experimental Animal Board.^9^ Both projects applied the same experimental hypoxic-ischaemic-neuroprotection protocol, were led by the same scientist, and used large white pigs.

Representative pigs from each study were selected based on comparable insult severity, as assessed by duration of low-amplitude electroencephalogram (EEG) <7 microvolts (μV) to match injury severity, with a minimum duration of 1200 s (20 min). Animals with premature death (survival <72 h) were excluded from the analysis. As halothane anaesthesia was administered in both studies during the hypoxic-ischaemic insult, the terms ‘awake’ and ‘anaesthetised pigs’ refer merely to anaesthesia administered during the post-insult treatment.

### Animal preparation

Supplementary Figure S1 shows a flowchart of the experimental design for this study. The pigs were kept at a physiological temperature for this species (38.5–39.0°C)^27^ and bottle-fed with pig formula *ad libitum* after transportation. The pigs were administered anaesthesia gas mixture in a closed box before tracheal intubation followed by umbilical vessel cannulation for fluid (dextrose 5% in NaCl 0.45% [5 ml kg^−1^ h^−2^]), drug administration and continuous mean arterial blood pressure (MAP) recording. Transcutaneous arterial O_2_ saturation (Tc*S*ao_2_) was measured by a pulse oximeter. Thirty minutes before hypoxia-ischaemia, in both studies, halothane concentration was adjusted to maintain end-tidal halothane concentration at 1% and end-tidal CO_2_ 4.5–5.0 kPa during baseline before the insult. Core temperature was recorded with a rectal thermometer at 6 cm depth and maintained at 38.5–39.5°C with an overhead heater Two-channel EEG signals were continuously recorded from subcutaneous needle electrodes over each hemisphere (interelectrode distance 3 cm). The baseline EEG was recorded for 60 min before, during, and after the hypoxic-ischaemic insult.

### Acute global hypoxic-ischaemic insult

Acute hypoxia was induced by reducing fractional inspired O_2_ (*F*io_2_) to 4–7% pending the EEG voltage response. The inspired halothane concentration was reduced to 0.6–0.8% pending the MAP response to achieve controlled hypotension <40 mm Hg. The *F*io_2_ and inspired halothane concentration were continuously titrated to maintain a low-amplitude EEG (peak-to-peak amplitude <7 μV) whilst avoiding severe hypotension (MAP <30 mm Hg) and bradycardia (<100 beats min^−1^). When hypotension or bradycardia occurred, the *F*io_2_ was transiently increased, halothane decreased, or both until the recovery of blood pressure and HR. Severe bradycardia that was unresponsive to increased *F*io_2_ or cardiac arrest were treated with 100% *F*io_2_ and cardiac compressions. The hypoxic-ischaemic insult was terminated after 45 min and pigs were reoxygenated with O_2_ 100% until the Tc*S*ao_2_ reached ≥95%,^9^ which was the clinical practice at the time. The anaesthetised pigs were first reoxygenated in air and then with the minimal *F*io_2_ required to achieve ≥95% Tc*S*ao_2._^15^

### Post-insult treatment

In the awake pigs, anaesthesia was stopped upon reoxygenation. Mechanical ventilation was terminated when the pigs were able to breathe independently. In the anaesthetised pigs, inhalation anaesthetics were replaced by i.v. anaesthetics (propofol 4 mg kg^−1^ bolus followed by propofol 4–12 mg kg^−1^ h^−2^ and remifentanil 20–80 μg kg^−1^ h^−2^), with the dose adjusted according to clinical need (HR, MAP, responsiveness).

Immediately after reoxygenation, the pigs were randomly allocated to treatment with normothermia or hypothermia for 24 h. During treatment, the awake pigs were kept unrestrained in a closed neonatal temperature-controlled incubator. All intravascular lines and the deep rectal temperature probe were taped along the flank and onto the back.

In the awake pigs, the rectal temperature was maintained at 39.0°C during normothermia or 35°C during hypothermia. In the anaesthetised pigs, the rectal temperature was maintained at 38.5°C during normothermia or 33.5°C during hypothermia. After 24 h of hypothermia, pigs were rewarmed to 38.5–39°C rectal temperature over a period of 6–10 h.

### Histological processing and classical histopathology

After survival for 72 h, the pigs were euthanised under anaesthesia with halothane (awake pigs) or isoflurane (anaesthetised pigs). The brain was perfusion-fixed with phosphate 4%-buffered paraldehyde, paraffin-embedded, and sectioned coronally fronto-caudally into 15 blocks. Histological sections of 5 μm thickness were cut from each block and stained with haematoxylin and eosin (H&E) and immunohistochemistry (as described below).

The injury severity and distribution in the cerebral cortex, hippocampus, thalamus, basal ganglia, and cerebellum were evaluated on H&E-stained sections by pathologists EML and HP who were blinded to treatment and clinical severity. Each region was graded with a pathology score ranging from 0.0 to 4.0^27^ with intervals of 0.5. A score of 0 indicates no visible injury; a score of 1 indicates the presence of individual dead neurones or patchy areas of infarcts; a score of 2 indicates partly confluent infarctions; a score of 3 indicates large, complete infarcts; a score of 4 indicates complete disintegration of the tissue.^27^ The assessment was based on the presence of cell death, gliosis, and injury size on H&E-stained sections. The global pathology score is the average score of all assessed regions.

### Immunohistochemistry

Histological sections of 5 μm thickness from blocks 6–7 containing the hippocampus and blocks 11–12 containing the cerebellum were analysed with brightfield immunohistochemistry. Sections were deparaffinised at 60°C for 1 h, incubated for 15 min in xylene 100%, followed by graded rehydration in 5-min steps in ethanol and distilled water. Sections were washed in a solution of Tween-20-phosphate buffer 0.1% (0.1% PBS-T), followed by antigen retrieval by boiling at 96°C for 25 min in citrate buffer 10 mM (pH=6.0). Dual enzyme block (Dako, S2003, Agilent, Santa Clara, California, USA) was applied for 10 min to block endogenous peroxidases and phosphatases. All sections were incubated for 1 h in horse serum 10% in PBS-T 0.2%, after which primary antibodies were applied.

Sections from blocks 6–7 were incubated with anti-ionised calcium-binding adapter molecule 1 (IBA1) (CAE1308, Wako, Osaka, Japan) against microglia for 48 h at 4°C in horse serum 10% + bovine serum albumin (BSA) 5% in PBS-T 0.2%. Sections from blocks 11–12 were incubated with anti-Calbindin (c9848, #22190224, Sigma, St. Louis, Missouri, USA) against cerebellar Purkinje cells and anti-IBA1 in horse serum 10% + BSA 5% in PBS-T 0.2%. For the analysis of apoptosis, sections from blocks 6–7 were incubated with antibodies for cleaved caspase-3 (AB3623, Merck Millipore, Darmstadt, Germany), and sections 11–12 were incubated with antibodies for cleaved caspase-3 and calbindin. The cleaved caspase-3 antibody was validated using a positive control (pig spleen) and a negative control (without primary antibody).

After 48 h, the sections were washed in PBS-T0.1% and secondary antibodies were applied (horse-anti-rabbit HRP [brown] + horse-anti-rabbit AP [magenta]) with the Immpress Duet Double Staining Polymer kit (MP-7724, Vector Laboratories, Burlingame, California, USA). DAB and AP chromogens were applied sequentially. Haematoxylin was used for counterstaining. Lastly, stepwise dehydration followed before mounting with permanent mounting medium (DPX). After the sections had dried, they were scanned using a Zeiss Axioscan Z1 slide scanner at pixel resolution 0.220 μm×0.220 μm, then pre-processed in Zen-Lite and Fiji for image analysis as below.

### Cell analysis

For the analysis of cerebellar Purkinje cells and granular cells, 8–10 representative areas (counting frame area=1500×1500 μm^2^) within the cerebellar cortex were selected from the vermis and lateral cerebellar hemispheres. Separate counting of calbindin-positive cells, cleaved caspase-3 positive Purkinje cells, and cleaved caspase-3-positive granular cells were performed manually in ImageJ facilitated by the ‘*cell counter*’-plugin. The cell density (cells mm^−2^) was calculated by the following formula: density= $\frac{\left(average\;cell\;count\right)*\hat{10}6}{\left(1500*1500\right)}$*.* Likewise, 4–5 representative hippocampal areas (counting frame area=600×600 μm^−2^) were selected from CA1–CA4. Manual counting of cleaved caspase-3-positive pyramidal cells was performed.

Microglial reactivity was assessed in the cerebellar cortex, cerebral cortex, and hippocampus using morphological grading. Microglia were graded based on their somal size, ramifications, and density using a 6-point score ranging from 0.0 to 2.0, with resting microglia exhibiting a small soma, thin ramified processes, and sparse density, whereas reactive microglia were rounder with thicker and shorter processes or an amoeboid larger cell body with very few ramifications.^28^^,^^29^ Global microglial reactivity was calculated by averaging over all regions assessed.

### Statistics

Statistical analysis was performed in STATA (v.17.0, StataCorp LLC, College Station, Texas, USA) and GraphPad Prism 9 (GraphPad Software, La Jolla, CA, USA). Non-parametric statistics were applied, such as the Wilcoxon Mann–Whitney Kendall's tau-b. Inter-rater variability was assessed with Cohen's kappa. All values are provided as median (inter-quartile range [IQR]). Confidence intervals at 95% level (95% CI) are supplied.

## Results

A total of 18 awake (normothermia *n*=11, hypothermia *n*=7) and 14 anaesthetised (normothermia *n*=7, hypothermia *n*=7) pigs were included in this study. The median (IQR) weight was 1.68 kg (1.49–1.81 kg), with no differences between the groups. The median (IQR) age was 30 h (21–36 h) in the awake study and 23 h (12–31 h) in the anaesthetised study, with no difference between the treatment groups.

**Fig 1:**
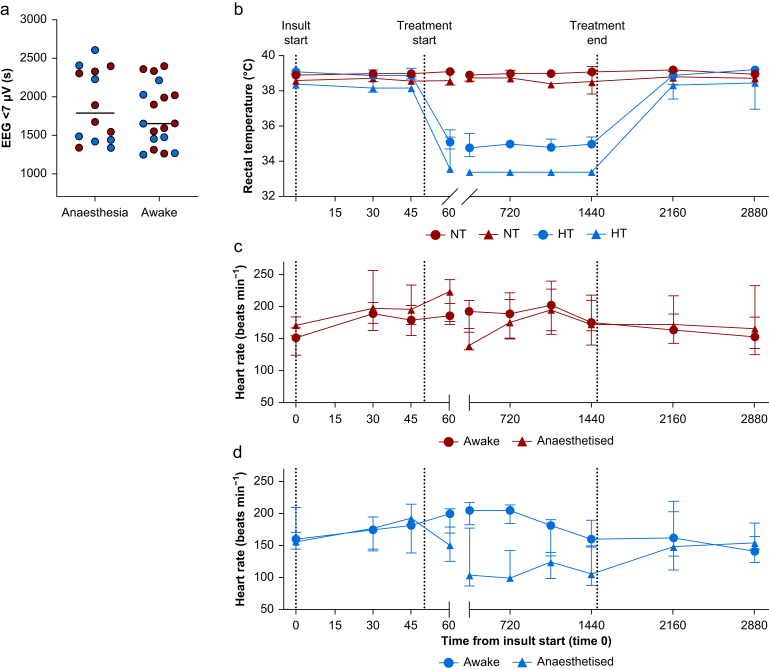
Physiological recordings. (a) Duration of low-amplitude EEG <7 μV was used to match injury severity across the two studies (anaesthesia *vs* awake). (b)–(d) Show recordings of rectal temperature (b) for anaesthetised (triangles) or awake (circles) pigs, heart rate for normothermia (NT), (c) and hypothermia (HT) (d). During hypothermia, heart rate was significantly greater in pigs that were awake during cooling compared with being anaesthetised.

The duration of low-amplitude EEG <7 mV was 31.6 min (25.8–38.8 min), 24.8 min (23.7–40.3 min), 31.7 min (25.9–38.9 min), and 24.7 min (21.2–33.9 min) in anaesthetised normothermia, anaesthetised hypothermia, awake normothermia, and awake hypothermia pigs, respectively (Fig. 1). The seizure burden was comparable between groups (∼30–40%), which is a similar incidence to that previously reported.^9^^,^^14^

**Fig 2:**
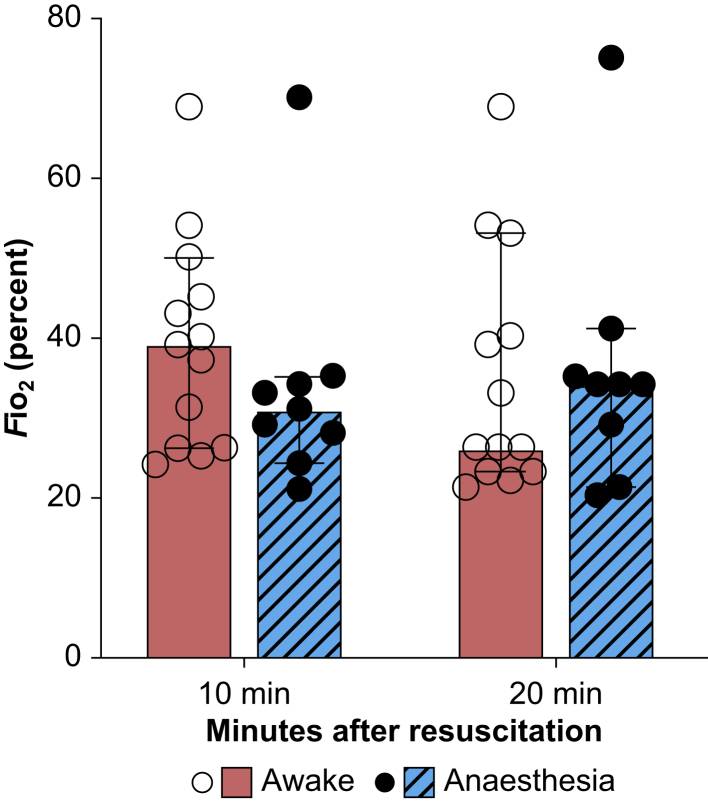
Reoxygenation fractional inspired O_2_. Distribution of *F*io_2_ at 10 and 20 min after insult. The two studies differenced in their protocol for reoxygenation. In the awake study, pigs were reoxygenated in O_2_ 100% as was the clinical practice at the time. By the time of the anaesthetised study, the clinical practice had moved towards resuscitation in air. Despite this difference, the level of *F*io_2_ at 10 and 20 min post-insult was comparable between the groups, indicating that that time of reoxygenation (and thereby difference in hyperoxia exposure) likely is negligible.

Although the resuscitation protocol differed between the studies regarding *F*io_2_ (starting with O_2_ 100% or air), the measured *F*io_2_ values at 10 and 20 min after reoxygenation were similar (Fig. 2)*,* indicating that the actual difference in exposure to hyperoxia likely was negligible. Physiological recordings of rectal temperature, HR, MAP, pH, and base excess during baseline, insult, and treatment are shown in Table 1 and Fig. 1. During hypothermia, awake pigs had a significantly higher HR compared with anaesthetised pigs at 6 h of treatment (*P*=0.008, 95% CI [22–123]), 12 h of treatment (*P*=0.005, 95% CI [37–116]), and 24 h of treatment (*P*=0.038, 95% CI [2–91]), which normalised 24 h after rewarming (*P*=0.69, 95% CI [−54 to 98]).

**Table 1:** Physiological recordings. Physiological data of rectal temperature, cardiovascular and metabolic changes at different timepoint during hypoxia, treatment, and recovery. The awake pigs undergoing hypothermia had significantly greater heart rate throughout the cooling period, compared with pigs remaining anaesthetised during hypothermia. HT, hypothermia; MAP, mean arterial blood pressure; NT, normothermia.

		Anaesthetised study				Awake study				Kruskal–Wallis
		NT, *n*=7		HT, *n*=7		NT, *n*=11		HT, *n*=7		
Baseline	Rectal temperature (°C)	38.6	(38.4–38.9)	38.4	(38.4–38.6)	38.9	(38.9–39.0)	39.1	(39.0–39.2)	0.001
	MAP (mm Hg)	47.0	(42.5–49.5)	54.0	(41.0–57.5)	58.0	(51.0–60.0)	54.0	(49.0–62.0)	0.126
	Heart rate	171.0	(158.5–178.5)	157.0	(154.5–185.5)	151.0	(126.0–160.0)	160.0	(148.0–165.5)	0.194
	pH	7.5	(7.5–7.6)	7.4	(7.4–7.5)	7.5	(7.4–7.5)	7.4	(7.4–7.5)	0.157
	Base excess	5.0	(−1.5 to 6.8)	3.0	(2.0–5.0)	5.0	(3.0–7.3)	4.8	(4.2–5.2)	0.799
End of insult (45 min)	Rectal temperature (°C)	38.6	(38.4–38.8)	38.2	(38.2–38.6)	39.0	(38.9–39.2)	38.9	(38.9–39.2)	0.001
	MAP (mm Hg)	36.0	(23.0–38.0)	30.0	(29.5–32.5)	40.0	(33.0–47.0)	45.0	(37.5–54.0)	0.069
	Heart rate	196.0	(179.0–222.0)	193.0	(185.5–195.5)	179.0	(156.5–193.0)	181.0	(149.5–203.0)	0.502
	pH	7.0	(6.9–7.1)	7.1	(7.0–7.2)	7.0	(6.9–7.1)	6.9	(6.9–7.0)	0.177
	Base excess	−20.0	(−23.0 to −17.5)	−17.0	(−22.0 to −14.5)	-23.0	(−27.5 to −19.9)	−25.6	(−27.1 to −25.2)	0.017
Recovery 6 h	Rectal temperature (°C)	38.8	(38.7–38.8)	33.4	(33.4–33.4)	38.9	(38.8–39.0)	34.8	(34.3–35.3)	<0.001
	MAP (mm Hg)	44.0	(41.0–48.0)	48.0	(46.5–50.5)	75.0	(64.5–92.0)	82.0	(67.0–88.0)	<0.001
	Heart rate	140.0	(134.0–163.5)	104.0	(93.0–163.5)	193.0	(162.0–206.0)	205.0	(191.0–210.0)	0.001
	pH	7.4	(7.4–7.5)	7.2	(7.2–7.3)	7.3	(7.3–7.4)	7.3	(7.3–7.3)	0.030
	Base excess	−6.0	(−7.0 to −5.0)	–9.6	(−11.3 to −6.9)	−5.5	(−6.2 to −3.7)	−9.9	(−12.8 to −5.4)	0.457
Recovery 12 h	Rectal temperature (°C)	38.8	(38.7–39.0)	33.4	(33.4–33.5)	39.0	(39.0–39.1)	35.0	(34.8–35.1)	<0.001
	MAP (mm Hg)	45.0	(44.0–49.5)	46.0	(43.0–50.0)	71.0	(67.5–85.0)	86.5	(81.5–92.2)	<0.001
	Heart rate	176.0	(154.0–211.5)	100.0	(97.5–134.5)	189.0	(162.5–210.5)	205.0	(190.2–213.0)	0.005
Recovery 24 h	Rectal temperature (°C)	38.5	(38.1–38.8)	38.6	(37.8–38.8)	39.1	(39.0–39.3)	35.0	(34.8–35.3)	<0.001
	MAP (mmHg)	54.0	(51.5–64.0)	48.0	(46.0–60.0)	65.0	(57.2–67.5)	69.5	(52.0–78.8)	0.196
	Heart rate	173.0	(167.0–196.5)	106.0	(92.0–140.5)	175.5	(145.8–204.0)	160.0	(151.5–178.5)	0.053
Recovery 48 h	Rectal temperature (°C)	38.8	(38.6–38.9)	38.5	(37.8–38.5)	39.0	(38.8–39.2)	39.2	(39.0–39.2)	0.031
	MAP (mm Hg)	75.5	(68.0–80.0)	60.0	(54.2–71.8)	61.0	(54.2–66.8)	70.0	(64.0–78.0)	0.431
	Heart rate	166.0	(145.0–205.5)	154.5	(154.0–155.8)	153.0	(131.2–180.0)	141.5	(126.8–172.8)	0.723

### Being awake during hypothermia abolishes neuroprotection

**Fig 3:**
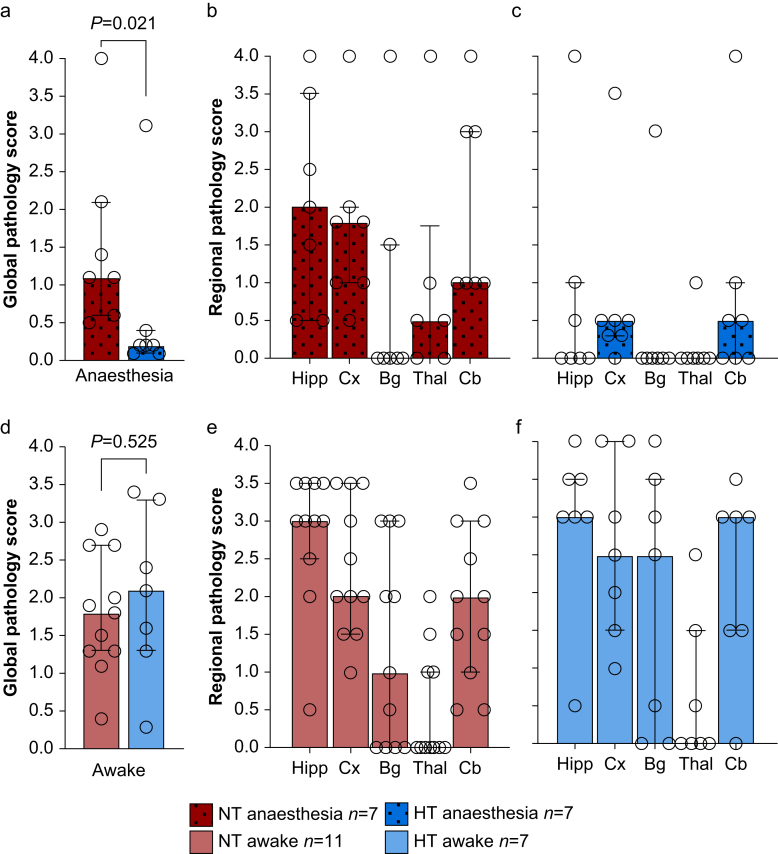
Classical histopathology. Global pathology score in (a) anaesthetised pigs and (d) awake pigs stratified by treatment group. Regional histopathology score is shown for (b) normothermia (NT) anaesthetised pigs, (c) hypothermia (HT) anaesthetised pigs, (e) normothermia awake pigs, and (f) hypothermia awake pigs. Hypothermia was neuroprotective among anaesthetised pigs, whereas neuroprotection was abolished in awake pigs. Wilcoxon Mann–Whitney statistics are presented. Bg, basal ganglia; Cb, cerebellum; Cx, cerebral cortex; Hipp, hippocampus; Thal, thalamus.

The inter-rater variability for histopathological analysis showed substantial agreement for the cortex (Cohen's kappa 0.636) and hippocampus (Cohen's kappa 0.739), and moderate agreement in the cerebellum (Cohen's kappa 0.463), using the kappa definitions in Cohen.^30^ Anaesthetised normothermia pigs achieved a median (IQR) global pathology score of 1.1 (0.8–1.7), with the hippocampus and cortex exhibiting the highest degree of injury (Fig. 3). The severity of injury was comparable between normothermia anaesthetised and normothermia awake pigs. In comparison, anaesthetised hypothermia pigs had a significantly lower global pathology score of 0.2 (0.1–0.3, *P*=0.021, 95% CI [0.30–1.95]), indicating effective neuroprotection by hypothermia compared with the anaesthetised normothermia group. Notably, anaesthetised hypothermia neuroprotection appeared most effective in the hippocampus and cortex (Fig. 3). However, in the awake group, hypothermia neuroprotection was completely abolished. The global pathology score was similar between normothermia (1.8; 1.3–2.7) and hypothermia (2.1; 1.5–2.9, *P*=0.525, 95% CI [−1.19 to 0.59]). In fact, comparing the pigs treated with hypothermia under anaesthesia *vs* those in the awake state, the extent of injury was nearly 10-fold greater in the latter group (*P*=0.021, 95% CI [0.15–3.15]).

### Greater cell death in CA1–CA4 of the hippocampus in awake pigs

**Fig 4:**
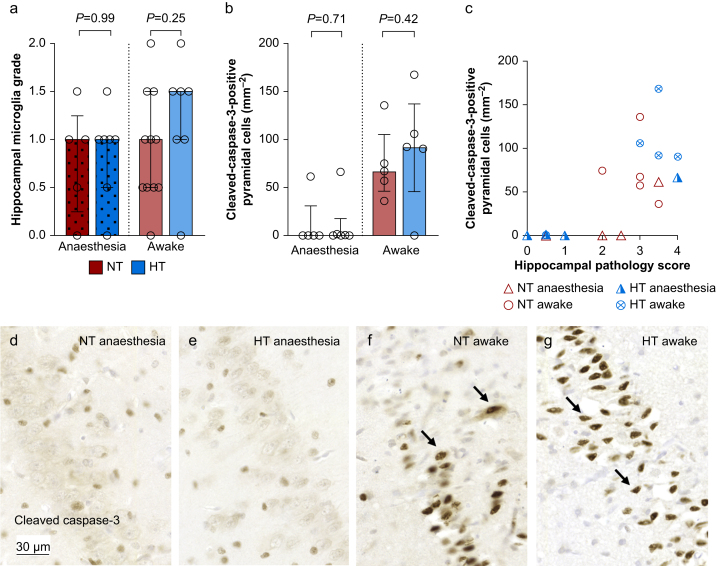
Hippocampus. (a) Microglial reactivity (image not shown) graded by morphological characteristics, assessed in the cornu ammoni of the hippocampus. (b) Density of pyramidal cells in CA1–CA4 positive for cleaved caspase-3. Wilcoxon Mann–Whitney statistics are presented. (c) Correlation between pyramidal CA1–CA4 cleaved caspase-3 density and hippocampal pathology score. (d)–(g) Show representative selection of pyramidal cells positive for cleaved caspase-3 in each group. HT, hypothermia; NT, normothermia.

We further investigated the hippocampus as it is a region with a high susceptibility to hypoxic-ischaemic injury. We did not observe a difference in microglial reactivity between the groups (Fig. 4). Interestingly, we detected increased numbers of cleaved caspase-3-positive pyramidal cells (indicating increased cell death) in CA1–CA4 of awake normothermia pigs compared with anaesthetised normothermia pigs (*P*=0.031, 95% CI [5.46–135.11]) but no significant difference between the hypothermia groups (*P*=0.062, 95% CI [−0.00008 to 167.4], Fig. 4). The density of cleaved caspase-3-positive pyramidal cells correlated with the pathology score (tau-b=0.526, *P*<0.001).

### Purkinje cell death is increased in awake pigs

**Fig 5:**
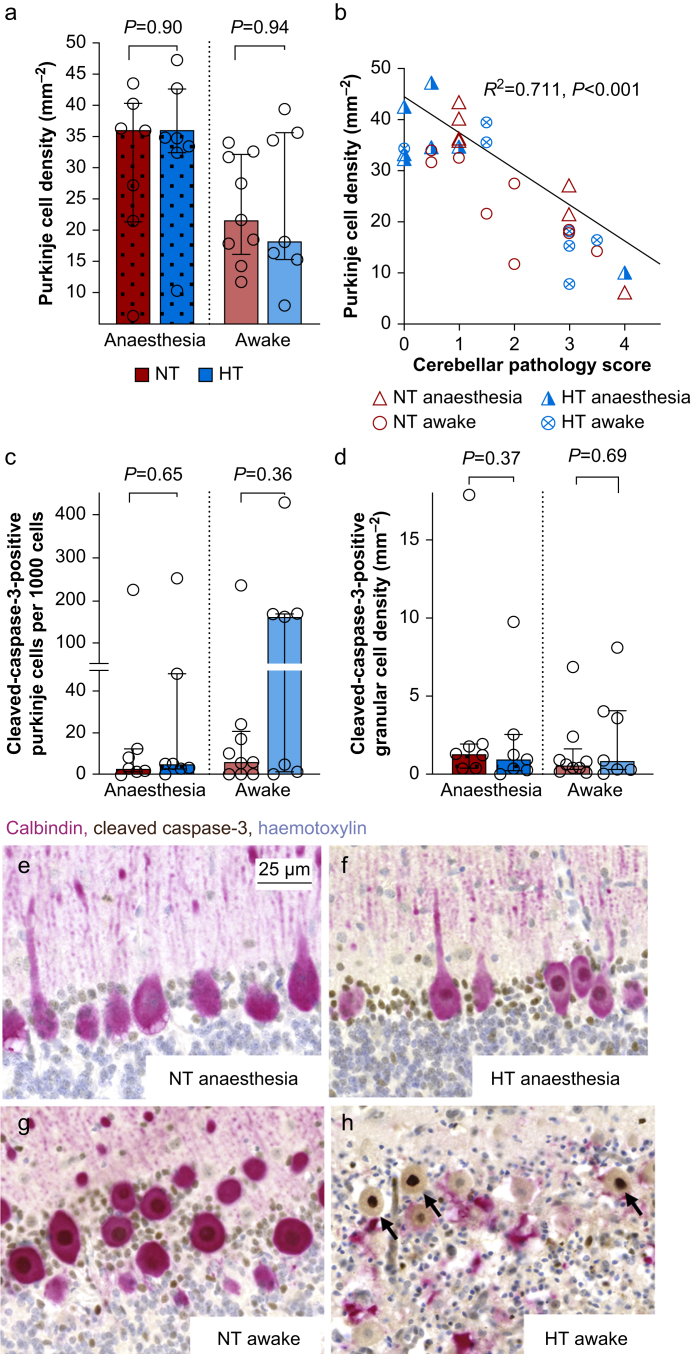
Cerebellum. (a) Bar graph of cell density of Purkinje cells. Wilcoxon Mann–Whitney statistics presented. (b) Correlation between Purkinje cell density and cerebellar pathology score. Kendall's tau-b presented. (c) Bar graph of ratio of positive cleaved caspase-3 pyramidal cells to all Purkinje cells. (d) Density of positive cleaved caspase-3 cells in the granular cell layer of the cerebellum. (e) Show representative selection of Purkinje cells positive for cleaved caspase-3 in each group. HT, hypothermia; NT, normothermia.

In the cerebellum, microglial reactivity was comparable across the groups. Purkinje cell density was not improved by hypothermia in anaesthetised pigs (normothermia 35.9; 21.4–40.2 *vs* hypothermia 34.6; 32.4–42.6, *P*=0.902, 95% CI [−15.93 to 13.76]) or awake pigs (normothermia 21.6; 16.0–32.1 *vs* hypothermia 18.2; 15.3–35.5, *P*=0.837, 95% CI [−20.12 to 8.78], Fig. 5). However, Purkinje cell density was lower in the awake group compared with the anaesthetised group, irrespective of treatment (*P*=0.034, 95% CI [−18.12 to −0.69]). Moreover, we observed a strong overall correlation between Purkinje cell density and cerebellar pathology score (tau-b=−0.572, *P*<0.001). Surprisingly, the number of cleaved caspase-3 positive Purkinje cells was greatly increased only in awake pigs receiving hypothermia (160.0/1000 cells; 1.4–168.0), corresponding to 16% of all Purkinje cells. Furthermore, we observed a strong correlation between the number of cleaved caspase-3-positive Purkinje cells and cerebellar pathology score (tau-b=0.496, *P*<0.001) and Purkinje cell density (tau-b=−0.530, *P*<0.001). However, the density of granular cells positive for cleaved caspase-3 was low and similar across all groups (Fig. 5).

## Discussion

We have explored the effects of anaesthesia on brain injury, neuroinflammation, and apoptosis in two distinct studies of global hypoxic-ischaemic injury in newborn pigs randomised to treatment of normothermia or whole-body hypothermia. Our study demonstrates increased injury in the pigs that remained awake throughout the 24-h hypothermia treatment period, with a loss of neuroprotection from hypothermia. This finding is specific to the newborn pig compared with other term-equivalent animal models of hypoxic-ischaemic encepaholpathy.^10^ Although the translatability of hypoxic-ischaemic hypothermia experiments in rodents is limited, it is noteworthy that loss of neuroprotection has not been reported in awake and unsedated postnatal day 7 rats or postnatal day 17 ferrets.^31^^,^^32^ This disparity may be attributed to physiological differences in heat-conserving thermoregulation,^33^ experimental differences in insult protocol, or both.

Based on our data, it is challenging to determine whether anaesthesia provides neuroprotection directly, through reduction of stress, or both. From previous work we know that pigs remaining awake and unsedated during hypothermia develop elevated plasma cortisol concentrations, reflective of an ongoing stress response during cooling.^9^ Anaesthesia mitigates this cortisol surge.^16^ During hypothermia, the HR is expected to decrease by 10 beats min^−1^ °C^−1^.^34^ In our study, the HR did not decrease during hypothermia in the awake pigs, being significantly higher than in cooled anaesthetised pigs (Table 1, Fig. 1) hence indicating a stress response. It is possible that the haemodynamic changes associated with stress, such as increased HR, contribute to the difference in injury susceptibility and neuroprotection.

There is evidence supporting direct neuroprotection from anaesthetics, such as propofol and remifentanil. Propofol has demonstrated neuroprotective effects *in vivo* through reduced Ca^2+^-induced mitochondrial swelling^35^ and suppression of excessive reactive oxygen species (ROS),^36^ which are important mechanisms involved in delayed cell death.^37^ In adult rats, propofol infusion attenuated infarct volume^22^ and improved Montoya staircase test performance after acute cerebral ischaemia.^38^ Remifentanil was found to reduce apoptosis in the gyrus dentatus and improve latency in the step-down avoidance task after transient global cerebral ischaemia in adult gerbils,^39^ and exert antiapoptotic and protective effects against ROS in postnatal day 2 mice.^23^

The two studies assessed here are suitable for comparative analysis because they share a similar protocol in terms of injury induction, duration, and mode of hypothermia (i.e. immediate onset, whole body, 24 h duration), post-insult management, and survival. Notably, clinical practice in neonatology with respect to reoxygenation and resuscitation changed between the earlier (awake) and later (anaesthetised) study. The awake pigs were resuscitated in 100% O_2_, which has been shown to induce excessive ROS formation, exacerbate hypoxic-ischaemic injury,^40^ and abolish hypothermia neuroprotection.^41^ The anaesthetised pigs were initially resuscitated in air followed by increasing *F*io_2_ as clinically indicated. This difference could potentially alter ROS burden and thereby explain some of the difference in injury between the awake and anaesthetised groups. However, in our experiments the duration of reoxygenation lasted only a few minutes, and *F*io_2_ stabilised at comparable levels between the two studies after only 10 min (Fig. 2). Therefore, we are confident our results primarily reflect the difference arising from 24 h of anaesthesia rather than the brief difference in *F*io_2_ during reoxygenation.

Another noteworthy difference between the studies is the discrepancy in target rectal temperature in the hypothermia groups. The awake pigs were cooled 4°C from 39.0 to 35.0°C core temperature, which yields a similar relative temperature reduction as used clinically (−3.5°C reduction from physiological temperature). The anaesthetised pigs were cooled from 38.5 to 33.5°C core temperature, which is the target temperature used clinically, however yielding a greater relative temperature reduction of −5°C. Whether it is best to induce the target temperature or the temperature reduction for translational studies remains unclear.^42^ However, cooling to either 35°C or 33.5°C after this global insult has previously shown similar neuroprotection and systemic effects in pigs.^42^^,^^43^

We were especially interested in studying the effect of anaesthesia on cell death, as our group previously demonstrated increased apoptosis in the granular cell layer after continuous fentanyl infusion in healthy newborn pigs.^24^ We used cleaved caspase-3 as a marker of cell death on the apoptosis–necrosis continuum.^44^^,^^45^ We unfortunately did not investigate the role of caspase-independent apoptosis and necroptosis^37^ specifically. We found no evidence to suggest that propofol–remifentanil induces cell death in our model. Interestingly, we observed an increase in cleaved caspase-3 cells in the awake hypothermia pigs. These findings could provide an explanation for the difference in injury and neuroprotection observed in this group.

## Conclusion

Our study provides novel insights into the significance of anaesthesia/sedation during hypothermia for achieving optimal neuroprotection. While the direct transferability of anaesthesia during hypothermia to sedation in clinical practice may be limited, our findings highlight the potential impact of sedation on mitigating stress-related cell death. These findings emphasise the need for further studies on analgesia/sedation/anaesthesia in the clinical setting to improve outcomes after hypoxic-ischaemic encephalopathy.

## Author’s contributions

All authors fulfil the ICMJE-criteria.

Study conception and study design: JKG, MT

Data collection: JKG, EC, DAM, MT

Analysis and interpretation of the results: JKG, EC, DAM, LW, MT

Draft manuscript preparation: JKG

Manuscript revision for important intellectual content: JKG, EC, DAM, LW, MT

Reviewed the results and approved the final version of the manuscript: all authors

Agree to be accountable for all aspects of the work: all authors

## Declarations of interest

All authors declare that they have no conflict of interest.

## Funding

The Research Council of Norway (NFR)FRIPROBIO ES492246, SPARKS UK (The Children's Medical Research Charity) 12LEO01, GOSH UK 18BTL01, 10.13039/501100005366University of Oslo Start-Up grant.

## Figures and Tables

**Fig. S1:**